# A zero coronary artery calcium score in patients with stable chest pain is associated with a good prognosis, despite risk of non-calcified plaques

**DOI:** 10.1136/openhrt-2018-000945

**Published:** 2019-04-11

**Authors:** Xue Wang, Elizabeth Phuong Vi Le, Nikil K Rajani, NJ Hudson-Peacock, Holly Pavey, Jason M Tarkin, Judith Babar, Michelle Claire Williams, Deepa Gopalan, James H F Rudd

**Affiliations:** 1 Division of Cardiovascular Medicine, University of Cambridge, Addenbrooke's Hospital, Cambridge, UK; 2 Department of Clinical Radiology, Imperial College Hospitals NHS Trust, St Mary's Hospital, London, UK; 3 Cambridge Clinical Trials Unit, Cambridge University Hospitals NHS Foundation Trust, Addenbrooke's Hospital, Cambridge, UK; 4 Department of Radiology, Addenbrooke's Hospital, Cambridge, UK; 5 Centre for Cardiovascular Sciences, University of Edinburgh, Edinburgh, UK

**Keywords:** coronary artery disease, chest pain clinic, CT scanning, risk stratification

## Abstract

**Objectives:**

To estimate the prevalence of non-calcified coronary artery disease (CAD) in patients with suspected stable angina and a zero coronary artery calcification (CAC) score, and to assess the prognostic significance of a zero CAC in these symptomatic patients.

**Methods:**

In this prospective cohort study, consecutive patients with stable chest pain underwent CAC scoring ± CT coronary angiography (CTCA) as part of routine clinical care at a single tertiary centre over 7 years. Major adverse cardiac event (MACE) was defined as cardiac death, non-fatal myocardial infarction and/or non-elective revascularisation.

**Results:**

A total of 915 of 1753 (52.2%) patients (mean age 56.8 ± 12.0 years; 46.2% male) had a zero CAC score. Of the 751 (82.1%) patients with a zero CAC in whom CTCA was performed, 674 (89.7%) had normal coronary arteries, 63 (8.4%) had non-calcified CAD with < 50% stenosis and 14 (1.9%) had ≥ 50% stenosis in at least one coronary artery. The negative predictive value of a zero CAC for excluding a ≥ 50% CTCA stenosis was 98.1%. Over a median follow-up period of 2.2 years (range 1.0–7.0 years), the absolute annualised rates of MACE were as follows: zero CAC 1.9 per 1000 person-years and non-zero CAC 7.4 per 1000 person-years (HR 3.8, p = 0.009). However, after adjusting for age, gender and cardiovascular risk factors using a multivariable Cox proportional hazards model, there was no statistically significant difference in the risk of MACE between the two patient cohorts (p = 0.19). After adjusting for age, gender and cardiovascular risk factors, the HR for all-cause mortality among the zero CAC cohort vers non-zero CAC was 2.1 (p = 0.27).

**Conclusion:**

A zero CAC score in patients undergoing CT scanning for suspected stable angina has a high negative predictive value for the exclusion of obstructive CAD and is associated with a good medium-term prognosis.

**Key questions**

**What is already known about this subject?**

- Many existing studies have looked at the prognostic value of a zero calcium score in asymptomatic study populations which have different prior probabilities of risk of major adverse cardiac event compared with symptomatic patient populations.

**What does this study add?**

- This study has the advantage of being a prospective study and looks at the medium-term prognosis of the symptomatic patient population with a zero calcium score.

**How might this impact on clinical practice?**

- The discovery of underlying coronary artery disease in those with zero calcium scores highlights the advantages of using CT coronary angiography in all subjects rather than having calcium scoring as a gatekeeper, as was the case in the previous version of the National Institute for Health and Care Excellence CG95 guideline.

## Introduction

Coronary artery calcification (CAC) is a well-established marker of future cardiovascular risk.^1^ Cardiac CT is widely used for the evaluation of stable coronary artery disease (CAD) due to it being a non-invasive, cost-effective and highly sensitive technique.^2^ CT calcium scoring detects and quantifies CAC and coronary CT coronary angiography (CTCA) allows for detailed anatomical evaluation of luminal stenosis secondary to both calcified and non-calcified atherosclerotic plaques.^2^

The absence of CAC (defined as a zero CAC score on CT) in asymptomatic individuals is associated with a very low incidence of cardiovascular events over a 15-year follow-up.^3^ However, 1%–2% of symptomatic patients with chest pain, and a zero CAC score, have non-calcified coronary artery atherosclerosis.^4 5^ The long-term prognosis of these symptomatic patients with a zero CAC score remains unclear.^6^

In November 2016, the UK’s National Institute for Health and Care Excellence’s (NICE) Clinical Guideline 95 for the evaluation of patients with stable chest pain was issued as an update of their own 2010 Guideline.^2 7^ The update recommended the removal of CT calcium scoring from the investigation algorithm on the basis that a zero CAC score may still be associated with significant underlying CAD, particularly non-calcified plaque, instead suggesting CTCA for all patients presenting with suspected angina.

Therefore, the objective of the study was to estimate the prevalence of non-calcified CAD in patients presenting with stable angina and a zero CAC score undergoing CT scanning in our centre. The secondary objectives were to compare incidence rates of major adverse cardiac events (MACEs), and all-cause mortality, in patients with a zero CAC versus a non-zero CAC score.

## Methods

### Study design

In this prospective study, consecutive patients with a suspected new diagnosis of stable angina underwent CAC scoring ± CT coronary angiography (CTCA) as part of routine clinical care at Addenbrooke’s Hospital from November 2009 to October 2016. This was a service evaluation audit and it was approved by the hospital’s audit committee. Patients were excluded from the study if they were asymptomatic, had prior diagnosis of CAD, myocardial infarction, percutaneous coronary intervention or coronary artery bypass grafting surgery. Patients referred for a cardiac CT scan for non-coronary indications such as arrhythmias, aortic valve and pericardial evaluations were excluded.

Clinical data were obtained from the hospital electronic patient record system. Hypertension was defined by a physician diagnosis of hypertension or the use of anti-hypertensive medications. Diabetes was defined by a physician diagnosis of diabetes or the use of anti-diabetic medications. Obesity was defined by a body mass index of 30 or greater. A positive smoking history was defined by the patient either being a current smoker (being an active smoker at the time of scan) or being an ex-smoker. A family history of premature CAD was defined as any first-degree relative with a history of relevant heart disease before age 60 or based on having a recorded family history of CAD in the patient record.

### CT imaging

Imaging was performed using a Siemens Flash 64×2-slice dual source CT scanner as per standard clinical protocols. CAC scoring was performed using the Agatston method^8^ with Siemens SyngoVia analysis software V.VB9 and V.VB10. Prior to CTCA imaging, intravenous metoprolol (5–20 mg) and sublingual glyceryl trinitrate (two sprays, 400 μg) were administered to all patients without contraindication. Intravenous contrast (70 mL Omnipaque 350) was administered at 6 mL/second followed by 40 mL 0.9% saline flush. CTCA images were acquired with prospective ECG gating (70%), high-pitch single heart beat acquisition, retrospective mode or a combination as needed to obtain diagnostic image quality. Tube current was 150–300 mA and voltage 80–100 kV. CAC scanning and CTCA were performed on the same day. All CT scans were reported jointly by accredited cardiac radiologists and cardiologists. The severity of non-calcified CAD on CTCA was graded as follows: normal coronary arteries, non-obstructive (at least one <50% luminal diameter stenosis) and obstructive (at least one ≥50% luminal diameter stenosis).

### Patient follow-up

Patients were followed up prospectively using the hospital electronic patient record system.

The endpoint, MACE, was defined as cardiac death, non-fatal myocardial infarction and/or non-elective revascularisation. Additionally, the incidence of all-cause mortality was evaluated. Revascularisation was defined as percutaneous coronary intervention or coronary artery bypass grafting surgery. A diagnosis of myocardial infarction was verified by a cardiologist.

### Statistical analysis

Continuous variables are presented as mean±SD or median and IQR, and categorical variables as counts with proportions. Comparison of between-group continuous variables was performed using the unpaired t-test, whereas the Pearson χ^2^, two-tailed test was used for comparison of categorical variables.

A survival analysis was conducted with individual subject time-to-MACE using the Kaplan-Meier method, with curves representing MACE-free survival according to zero CAC or non-zero CAC. Annualised rates of MACE and all-cause mortality were estimated by dividing the number of events by the number of person-years at risk.

HR and 95% CIs were calculated with the Cox proportional hazards regression model using zero CAC as the reference group. Three models were constructed: Model 1: unadjusted, univariable analysis; Model 2: multivariable analysis adjusting for age and gender; Model 3: multivariable analysis adjusting for age, gender and cardiovascular risk factors (hypertension, diabetes, obesity, smoking history and family history).

All statistical analyses were performed using IBM SPSS Statistics for Macintosh, V.25.0. A two-tailed p value of <0.05 was considered statistically significant.

## Results

### Characteristics of Study cohort

**Figure 1:**
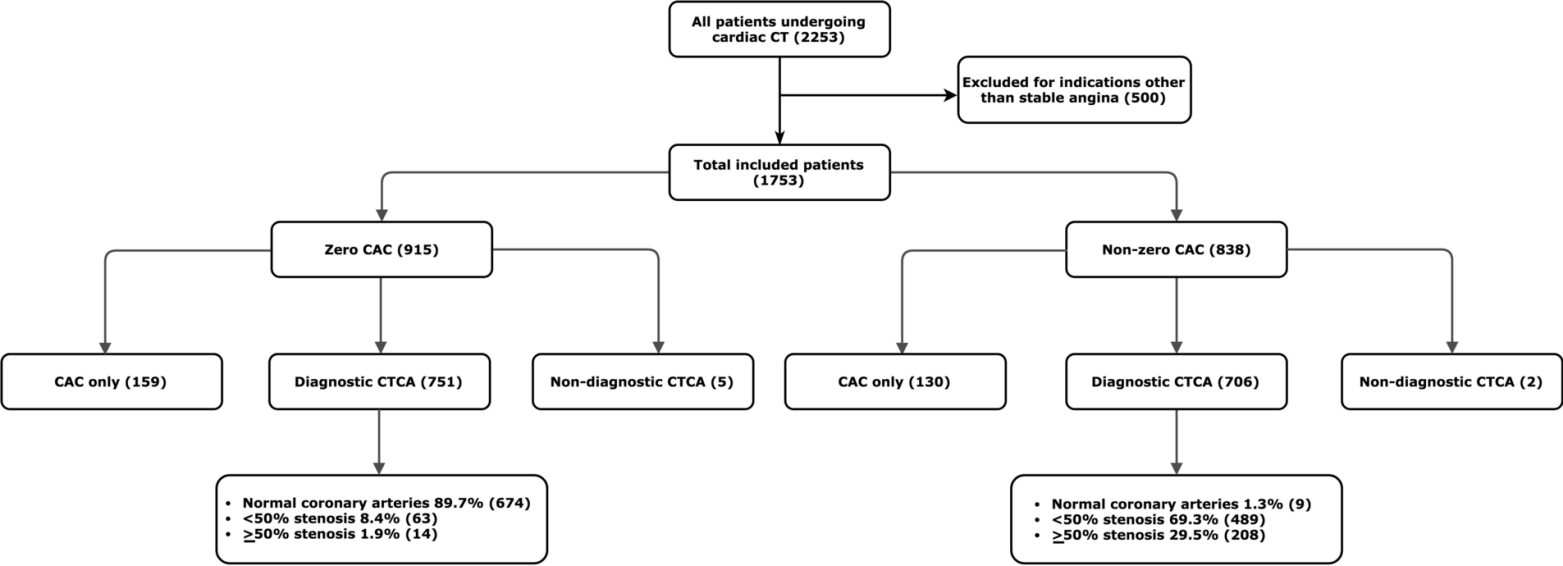
Study flow chart. The values in parentheses represent the numbers of patients in each part of the study. The percentages in the lowest boxes represent the fraction of patients in each disease category. CAC, coronary artery calcification; CTCA, CT coronary angiography.

Over a 7-year period, 2253 patients underwent cardiac CT imaging at our institution. In all, 500 patients were excluded from the study cohort as they underwent imaging for indications other than investigation of chest pain, or because of a non-diagnostic calcium scan. Therefore, 1753 patients with symptomatic chest pain (mean age 56.8±12.0 years) were included in the study (figure 1), and 46.2% were men. 52.2% of patients had a zero CAC versus 47.8% with non-zero CAC scores (median score 57.4 Agatston units; IQR 8.2–221). Patients with no detectable CAC were statistically more likely to be younger, women and less likely to have hypertension, diabetes or have a positive smoking history (table 1).

**Table 1:** Baseline characteristics of all patients and stratified according to CAC score

Variable	All patients (n=1753)	CAC=0 (n=915)	CAC >0 (n=838)	P value
Age (years), mean±SD	56.8±12.0	51.7±11.1	62.3±10.5	<0.0001
Male gender, N (%)	810 (46.2%)	360 (39.3%)	450 (53.7%)	<0.0001
Hypertension, N (%)	564 (32.3%)	198 (21.6%)	366 (43.7%)	<0.0001
Diabetes mellitus, N (%)	135 (7.7%)	40 (4.4%)	95 (11.3%)	<0.0001
Obesity, N (%)	211 (12.0%)	98 (10.7%)	113 (13.5%)	0.0746
Smoking history, N (%)	462 (26.4%)	219 (23.9%)	243 (29.0%)	0.0162
Family history, N (%)	559 (31.9%)	300 (32.8%)	259 (30.9%)	0.3989

CAC, coronary artery calcification;N, number.

A total of 289 patients did not undergo CTCA after CAC scoring (159 patients with a zero CAC and 130 with non-zero CAC). The decision to perform a CTCA after CAC scoring was in line with contemporaneous clinical guidelines and hospital protocol. The major reasons were first, a low pretest probability for CAD plus a zero CAC score (n=112), and second, extensive calcification on CAC scan thought likely to preclude CTCA analysis (n=88). CTCA was not performed in a further 89 patients because of difficult venous access, contrast allergy or patient preference. In addition, subjects with non-interpretable CTCA scans (n=7) were excluded from the CAD extent analysis, but their calcium scores and clinical progress contributed to MACE and mortality estimates.

**Figure 2:**
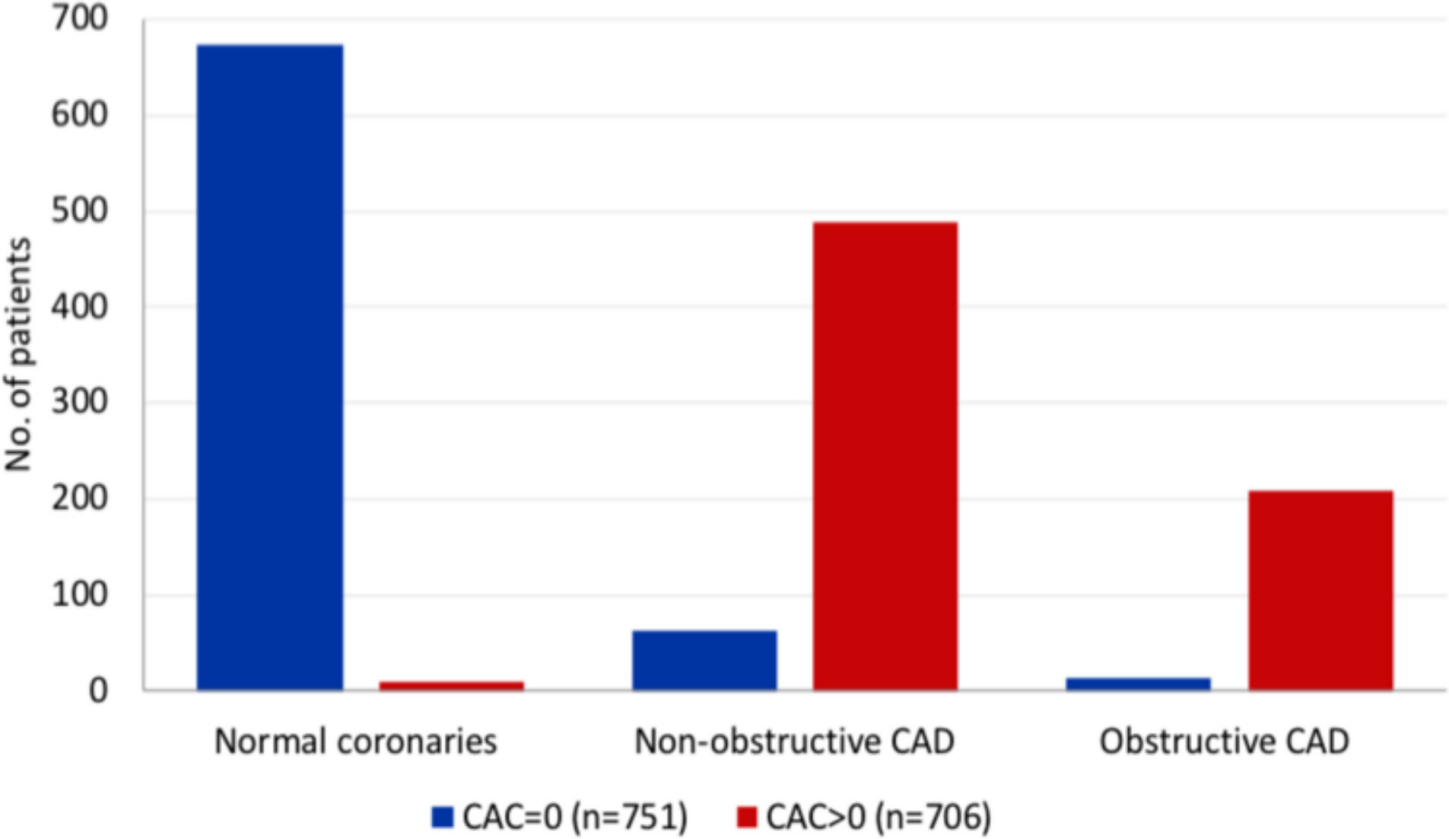
Prevalence of CAD following CTCA stratified according to CAC score. CAC, coronary artery calcification; CAD, coronary artery disease; CTCA, CT coronary angiography.

Of the 751 patients with a zero CAC in whom CTCA was performed, 674 (89.7%) had normal coronary arteries, 63 (8.4%) had <50% stenosis and 14 (1.9%) had ≥50% stenosis (figure 2). In the non-zero CAC cohort, of the 706 patients who underwent CTCA, 9 (1.3%) had normal coronary arteries, 489 (69.3%) had <50% stenosis and 208 (29.5%) had ≥50% stenosis.

In those with a zero CAC, there was no significant difference in the baseline risk factors in those with or without obstructive CAD (≥50% stenosis) with the exception of obesity and smoking history (table 2).

**Table 2:** Baseline characteristics of patients with a zero CAC and absence or presence of ≥50% stenosis on CTCA

Variable	CAD <50% (n=737)	CAD ≥50% (n=14)	P value
Age (years), mean±SD	51.73±11.24	54.49±8.13	0.3619
Male gender, N (%)	311 (42.2%)	8 (57.1%)	0.2624
Hypertension, N (%)	166 (22.5%)	5 (35.7%)	0.2436
Diabetes mellitus, N (%)	30 (4.1%)	1 (7.1%)	0.5670
Obesity, N (%)	71 (9.6%)	4 (28.6%)	0.0192
Smoking history, N (%)	165 (22.4%)	8 (57.1%)	0.0022
Family history, N (%)	230 (31.2%)	6 (42.9%)	0.3523

CAC, coronary artery calcification; CAD, coronary artery disease;CTCA, CT coronary angiography; N, number.

The negative predictive value of a zero CAC for excluding obstructive CAD (≥50% stenosis) was 98.1%. The sensitivity of this was 93.7% and the specificity was 59.7%.

### Follow-up and MACE

**Figure 3:**
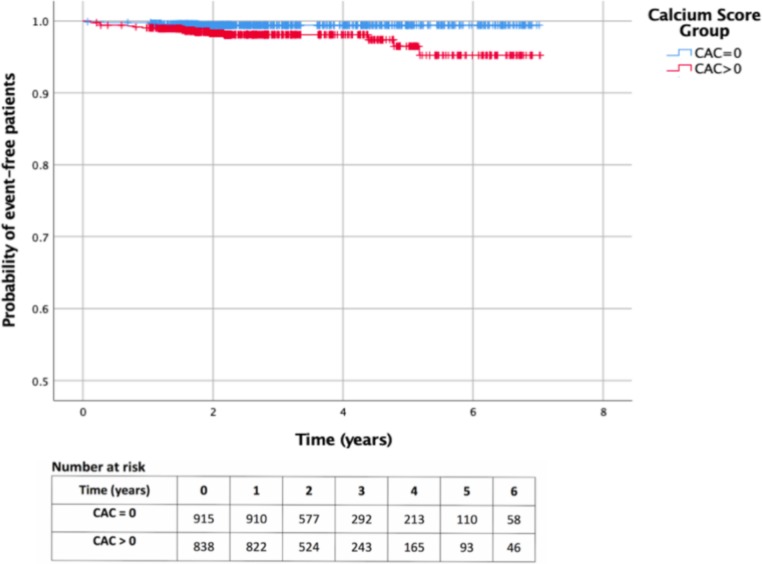
Kaplan-Meier curves stratified according to CAC score (total population). CAC, coronary artery calcification

Patients were followed up for a median of 2.2 years (IQR 1.8–3.7 years). The longest follow-up period was 7 years. Kaplan-Meier curves for MACE-free survival are presented in figure 3. The number of MACEs was 5 (0.6%) in the zero CAC cohort versus 17 (2%) in the non-zero CAC cohort. The annualised rate of MACE in the zero CAC cohort was 1.9 per 1000 person-years versus 7.4 per 1000 person-years in the non-zero CAC cohort (table 3).

**Table 3:** Annualised MACE rates for all patients and stratified according to CAC score

	No. of patients (%)*	No. of MACE (%)†	Rate/1000 person-years at risk
CAC=0	915 (52.2%)	5 (0.55%)	1.92
CAC >0	838 (47.8%)	17 (2.03%)	7.37
All patients	1753 (100%)	22 (1.25%)	3.46

*Proportion of all patients.

†Proportion within each group.

CAC, coronary artery calcification; MACE, major adverse cardiac event.

MACE in those with zero CAC were due to non-fatal myocardial infarction and/or non-elective revascularisation. None died of a coronary event. MACE occurred at a time interval of 13 days to 1.8 years after cardiac CT. Of the patients who had a zero CAC, three patients had normal coronary arteries and one patient had ≥50% stenosis on CTCA. Of note, 1 patient who had a zero CAC score did not have CTCA and subsequently developed a non-ST elevation myocardial infarction and received non-elective revascularisation at 116 days post-scan.

In the unadjusted univariable analysis, there was moderate evidence that the survival distributions for the zero CAC versus the non-zero CAC cohort differed (HR 3.8, 95% CI 1.4 to 10.3, p=0.009). After adjusting for the confounding factors age and gender, this difference was no longer statistically significant (HR 2.6, 95% CI 0.87 to 7.9, p=0.09). After adjusting for age, gender plus cardiovascular risk factors, the HR for MACE among the zero CAC cohort vers non-zero CAC was 2.1 (95% CI 0.69 to 6.5, p=0.19).

### All-cause mortality

During follow-up, the incidence of all-cause mortality was three patients (0.17%) in the zero CAC cohort versus 20 patients (1.1%) in the non-zero CAC cohort. Annualised rates of all-cause mortality for the zero CAC cohort were 1.2 per 1000 person-years vers 8.7 per 1000 person-years in the non-zero CAC cohort.

In the unadjusted univariable analysis of all-cause mortality, there was evidence that the survival distributions for the zero CAC versus the non-zero CAC cohort differed (HR 7.6, 95% CI 2.3 to 26, p=0.001). After adjusting for age and gender, this was no longer statistically significant (HR 2.5, 95% CI 0.67 to 9.1, p=0.18). After adjusting for age, gender plus cardiovascular risk factors, the HR for all-cause mortality among the zero CAC cohort versus non-zero CAC was 2.1 (95% CI 0.56 to 8.0, p=0.27).

### Composite endpoint: MACE and/or all-cause mortality

A composite endpoint comprising non-fatal myocardial infarction, non-elective revascularisation and/or all-cause mortality resulted in seven patients (0.40%) in the zero CAC cohort versus 36 patients (2.05%) in the non-zero CAC cohort with events. Following univariable analysis, there was evidence that the survival distributions for the zero CAC versus the non-zero CAC cohort differed (HR 5.8, 95% CI 2.6 to 13.0, p<0.0001). This difference remained statistically significant after adjusting for age and gender (HR 2.8, 95% CI 1.2 to 6.8, p=0.02). After adjusting for age, gender plus cardiovascular risk factors, this was no longer statistically significant (HR 2.3, 95% CI 0.9 to 5.7, p=0.065).

## Discussion

Studies in asymptomatic individuals have shown that the absence of CAC is a reliable screening tool in the evaluation for suspected CAD.^3 9^ However, there is uncertainty in the prevalence of CAD in symptomatic patients with a zero CAC score and in its longer-term prognostic implications.^10^

### Prevalence of non-calcified CAD in symptomatic patients with a zero CAC

In this prospective cohort study of patients with stable chest pain, a zero calcium score was associated with a low rate of CAD, with 8.4% subjects having non-obstructive disease and only 1.9% found to have obstructive disease. Furthermore, a zero score had a high negative predictive value of 98.1% for excluding obstructive CAD.

Our results agree with similar studies in symptomatic patient populations. Among patients with zero CAC in the COroNary CT Angiography Evaluation For Clinical Outcomes: An InteRnational Multicenter Registry (CONFIRM) registry, 13% had at least one <50% stenosis and 3.5% had at least one ≥50% stenosis.^10^ In a single-centre prospective cohort study of 1114 patients, the prevalence of ≥50% stenosis was 4.3%.^11^ Negative predictive values of 99.5%, 99% and 96%, respectively, have been quoted in the literature,^10 12 13^ close to our findings. The negative predictive value for zero CAC compares well with CTCA and stress imaging tests.^14^

### Prognostic significance of a zero CAC

Over a medium-term follow-up, the incidence of MACE and all-cause mortality in our patients was lower in the zero CAC cohort than the non-zero CAC cohort. In multivariable survival analysis after adjusting for age, gender and cardiovascular risk factors, there was no difference in the prognosis between the two cohorts for both MACE and all-cause mortality end points (p=0.19 and p=0.27, respectively). However, the overall prognosis of stable angina in patients with zero CAC was good with an annualised rate of MACE in the zero CAC cohort of 1.9 per 1000 person-years.

We compared both MACE and all-cause mortality in patients with zero CAC versus non-zero CAC, whereas previous studies have focused only on all-cause mortality, and showed this to be 0.05% to 3.8% over a mean follow-up period of 5.6–15 years.^3 9 12^ In another study, the incidence of MACE was found to be 1.3% over 2.8 years.^11^ Importantly, these studies align with our findings that the overall prognosis of patients with a zero CAC is largely unaffected by the presence of non-calcified atheroma.^12^

### CAC scoring versus CTCA

Although subjects with zero CAC had a good prognosis over during medium-term follow-up, 10.3% were found to have CAD on subsequent CTCA. Once identified, this group can benefit prognostically from medical therapy as demonstrated in the Scottish COmputed Tomography of the HEART Trial (SCOTHEART).^15 16^ The discovery of underlying CAD in those with zero calcium scores highlights the advantages of using CTCA in all subjects rather than having calcium scoring as a gatekeeper, as was the case in the previous version of the NICE CG95 guideline. CTCA without prior CAC scoring also lowers patient radiation exposure.

### Study limitations

As an observational study, clinical data were obtained from the electronic records system and there may have been incomplete patient follow-up. We assumed that loss to follow-up would occur equally across the study cohort, thereby not unduly influencing results. As a single-centre study, our findings may not be applicable in other geographic regions. Finally, while we analysed the degree of luminal stenosis on CTCA, we did not take into account the impact of high-risk plaque features that are also known to have prognostic significance.^17^

## Conclusion

A zero CAC score in patients with stable chest pain reliably excludes obstructive CAD and is associated with an overall very good prognosis. However, CTCA provides further diagnostic and prognostic information, revealing underlying CAD in over 10% of patients. We now know that such patients should be treated with preventive medical therapy to improve their prognosis.
